# Review of Parisian Medicine and Surgery

**Published:** 1860-02

**Authors:** Theophilus Mack

**Affiliations:** Lecturer on Materia Medica and Therapeutics in the Medical Department of the University of Buffalo


ART. II.—*Review of Parisian Medicine and Surgery.* By THEOPHILUS MACK, M. D., Lecturer on Materia Medica and Therapeutics in the Medical Department of the University of Buffalo.

The solemn Dons of Parisian Medicine have been anything but prolific in matter during the past three months. In the hospitals, at the scientific *séances*, and in the columns of the medical periodicals, what was interesting and instructive was old, and what was new, with a few exceptions, could scarcely be referred to one head or the other.

In the obstetrical department, M. Butignot makes a contribution upon the practice advisable in cases where the narrowness of the vulva gives rise to fear of laceration of the perineum. He commences by advancing the frequency of such laceration in primiparæ: stating that in the greater number of cases, the posterior commissure of the vulvar ring suffers in its integrity. The results of this accident are then expatiated upon, and the means hitherto devised for their counteraction. M. Eschilberg had recommended an incision at the lower part of one or both sides of the perineal raphe. This procedure meets with M. Butignot's approval, and he reports the following as a *model case :*

"Called to the accouchement of a young primiparous female, of small figure, of a slightly rachitic aspect, and with whom I apprehended an obstacle in the pelvis rather than in the soft parts. On the contrary, at first I had only to remain the spectator of a labor advancing with regularity, and my intervention had been so far useless, when, at the moment when I thought the accouchement was about to terminate, I remarked that, notwithstanding some energetic contractions, notwithstanding the dilatation of the anus, the extreme distention of the perineum, the vulva would not yield, and that a laceration was inevitable. What to do in such a circumstance? Could I dream of having recourse to the forceps? Its application, without doubt, was easy in the situation of the head; but should I thus obviate the danger? I thought not; I feared rather to increase it, and I decided to remove the constriction by one or two lateral incisions. Armed with a pair of good scissors, which usually serve me to divide the cord, and which, consequently, I had at hand, I introduced flatly one of the branches to the depth of about half an inch between the head of the infant and the right lateral and inferior part of the distended border of the vulvar orifice; next, turning the cutting edge towards this border, I made an incision, ready—as it appeared to me to be somewhat insufficient—to effect a second upon the opposite side: of this there was no necessity. Scarcely had the first been made when, without my being able to attribute it to the influence of a new contraction, by the sole effect of the retraction of the distended tissues, and without the least flow of blood, I perceived the angles of this incision growing round, disappear, and become confounded with the circumference of the vulva, which was thus found considerably enlarged, and at last the head of the infant escaped with the greatest facility, leaving the posterior commissure completely intact."

The result justified this operative interference; the convalescence was rapid and uninterrupted. One month after the accouchement there remained a little nucleus of induration only, which would most probably soon disappear, and the vulva was preserved in contour.—*Journal de Méd. de Toulouse.*

The importance of the subject of prolonged gestation, especially in legal medicine, need scarcely to be insisted upon; yet all that the profession can testify to, is by no means satisfactory in courts of law. The able article from the pen of Dr. Simpson, may be assumed to present a fair summary of our views upon the subject at present. The Caledonian professor, however, appears to have embraced the opinion most popularly received as to the duration of pregnancy to ten, twelve, or more months. It is quite probable that a closer scrutiny of the facts cited by authors relative to this exception to one of nature's laws, might lead to our recognition of much fewer of such cases, and limit the time we affix to possible duration of pregnancy.

M. Casper, Professor of Legal Medicine in the University of Berlin, after citing some of the cases reported, and showing how little worthy of credit they are, says, and here we shall allow him to speak for himself:—

"There is no doubt that pregnancy may last over the mean duration of 275 to 280 days. But physiology and observation accord in assigning it a certain limit. From the most ancient times, the cycle of ten menstrual periods has been considered as the duration of gestation; and it was Cederschgold who had the merit the first to have remarked that the interval between two menstruations is not the same with all women, and that, consequently, the duration of pregnancy may vary according to individual organizations. Thus we shall find with females who have their courses every 28 days, as most frequently happens, gestation lasts $28 \times 10 = 280$

days; with those who are regulated every 29 days, the pregnancy continues 29 × 10 = 290 days; lastly, with those who have their menses every 30 days, gestation goes to 30 × 10 = 300 days. M. Schuster has continued the observations of Cederschgold. He has communicated, in his article, four observations, of which two concerned his own wife. This lady had between her menstruations the interval of 29 to 30 days; the first pregnancy had lasted 296 days, and the second, 300 days. A healthy woman who had between her menstrual epochs an interval of about 29 days, was confined at one time after 287 days, and another time after 288 days.

"After what precedes, we conclude,—

"1. The ordinary duration of pregnancy is from 275 to 280 days.

"2. Gestation may, without any doubt, last longer; say to the 300th day.

"3. The longer terms, the tardy births of eleven, twelve, and thirteen months, are to be completely rejected. It follows that the laws have perfectly adopted the *terminus ad quum*, and that science does not demand any modification."

A simple method of ligating fibrous uterine polypi is communicated by Dr. Hamon. It may prove suggestive to some *confrère* who has not the different instruments employed, especially as they are instruments by no means of daily use.

The only apparatus consists of a female catheter and a piece of annealed iron wire. Near the mouth of the catheter, and at a point corresponding to the concavity of the instrument, a supplementary eye is made. The iron wire about 60 centimeters in length, has a series of knots, about 15 in number, at one of its extremities, about 8 millimetres apart; it is introduced into the catheter, and each of its two ends are engaged successively in the additional eye, the resulting loop constitutes the ligature for constriction. The simple extremity of the wire should be secured to the ring of the catheter to be permanent, the constriction is effected with the knotted end. The polypus being strangulated in the loop, the knotted extremity is very easily secured in the mouth of the sound by a tight-fitting peg of wood.

The lectures of M. Noel G. de Mussy, at the Hotel-Dieu, upon *circum-uterine phlegmasiæ*, would well repay the labor of translating *in extenso.*

The menstrual orgasm is considered the principal cause; one authentic observation of their occurrence after the "*menopause*" only existing. The most powerful occasional cause is abortion or parturition. The relative frequency of the different seats of the inflammation show, it is nearly 42 out of 50 times in the large ligaments; 14 out of 42 times, in the recto-uterine cul-de sac; 3 out of 42, in the utero-vesical space. According to the ancient opinion, the inflammation was seated in the infra-peritoneal cellular tissue. More recently, the peritoneum itself was assigned to it. The lecturer believes that the inflammation is readily propagated from the peritoneum to the subjacent cellular tissue. This cellular tissue becomes the site of serous infiltration, then purulent. The congestion may be sufficiently intense to produce ecchymoses. If the inflammation continue long acute, an abscess soon forms. Often the inflammation stops short of suppuration; and under the influence of a low chronic grade of irritation the cellular tissue, infiltrated with plastic lymph, becomes indurated, lardaceous, retracts, and then result displacements of the womb. In a second article we shall return to the general history of "*Circum-uterine Inflammations.*"

The ophthalmoscope is found to be capable of producing some mischievous results. It was certainly to be expected that injury to vision might occasionally follow the prolonged exposure of the eye to a strong light.

A case of contraction of the fingers resulting from the lesion of a nervous filament in venesection, was immediately cured in Sardinia by M. Borelli, by subcutaneous neurotomy; the division being effected at a point where a thread-like induration could be discovered near the superficial radial vein.

M. Vaquet has made the amputation of the foot below the astragalus, the subject of an inaugural thesis. Having described the various operative procedures, the advantages are thus summed up: "The sub-astragalus amputation offers excellent conditions for equilibrium, because the axis of the body falls perpendicularly upon the centre of the astragalus, which transmits it directly to the sole; walking and standing are perfectly assured, and the only question is to know if the inequalities of the calcanean surface do not fatigue the subjacent soft parts, and permit the repeated pressure which walking produces." These were the words of M. Ledillot in 1853. The observations of the author show that the amputated walk upon their stump without apparent lameness, if the boot be properly constructed. They all preserve the motions of the tibio-tarsal joint. His observations are sufficiently eloquent to display the advantages of this amputation above the others, when a flap from the plantar or *dorsal* aspect of the foot can be obtained, and when the state of the bones permits it.

In reference to a new method of operating for varicocele at the Hotel-Dieu, M. Jobert (de Lamballe) observes:—

"For some years I have had recourse to a new process of great simplicity, and the success of which has been constant. This process consists in compressing and in dividing the vessels."

The Professor's plan consists in engaging the varicose mass in a ligature of silver wire passed through the scrotum, and secured by passing through a heart-shaped metallic body, the ends being wound round two little knobs at the base. Now, we have, with a silk ligature, ourselves operated quite successfully in an exactly similar manner, viz., tying the veins, including a small portion of scrotal integument, having carefully excluded the cord by pushing it towards the symphisis pubis; and this mode of operating was taught by Prof. F. H. Hamilton fifteen years ago; but inasmuch as we were not present at the Gallic surgeon's clinique, we could not assert the claims of our friend to priority.

Another unsuccessful case of gastrotomy is reported in the *Gaz. Hebdom.* It was resorted to as a means of temporary relief in a case of dysphagia from cancer of the œsophagus.

M. Demarquay adopted a new method for the relief of deviation of the nasal septum resulting from a blow of the fist. "He made along the median line of the nose an incision, which, commencing at the back of this organ, ended upon the upper lip. The first stage of the operation separated the two lateral cartilages of the nose, and conducted to the median cartilage. This accomplished, M. Demarquay dissected to the left the mucous covering of the cartilage which filled the nostril; and when all the projecting part of this cartilage was thus exposed, he raised it, cutting from behind forward all that obstructed the nostril and hindered the respiration from being accomplished. This done, he reunited by points of suture, the divided lobule of the nose, and the affection was cured perfectly. The nose was straightened, and the respiration re-established upon the left as well as the right side. Reunion was accomplished by the first intention; and when the young man left the hospital there remained no more trace, either of the primary accident, or of the operation to which it had given place.

At the *Société Chirurgicale*, M. Chassaignac stated that he had ligated the primitive carotid under the following circumstances: A large fluctuating tumor at the base of the lower jaw gave rise to the suspicion of a retro-pharyngeal abscess; upon plunging a second time a bistoury into this, by the mouth, a jet of vermilion-colored blood filled the patient's mouth, so as to threaten suffocation. Various expedients having failed to arrest the flow of blood, the operation referred to was performed at the moment, and had proved successful. The cause of the accident, M. Chassaignac concluded to have been an anomalous distribution of the carotid; the diagnosis of an abscess was probably correct.

"*Academy of the Sciences.*" M. Matteucci announces that the electric current has no action upon a nerve, except along its length. He has also observed that the electromotor power is greatly diminished in the muscles of frogs killed by the *curare* poison.

M. Wurtz concludes from his researches that urea is discoverable in the lymph and chyle after the same manner as in the blood, from which he infers that the lymphatics contribute to the absorption of matter arising from the metamorphosis of the tissues into the midst of which these vessels plunge their radicles.

The theory of hepatic glycogenesis has received support from the experiments of M. C. Schmidt, of Dorpat, which show that in carnivorous animals, the blood entering the liver by the venæ portæ, is free of sugar, while the blood leaving the liver by the hepatic veins, contains notable quantities of this substance.

M. Tavignot obtains occlusion of the lachrymal ducts for the cure of fistula lachrymalis, by introducing a stilet of platina as far as the lachrymal sac, and heating it to a white heat by connection with a Bunsen's pile. The eschar which obstructs the ducts opposes itself immediately to the passage of the tears, and when this is eliminated, the cicatrix which has formed has already obliterated these same ducts.

M. Billard has found chlorate of potash, as an external application, to destroy effectually the odor from gangrene.

At the "Academy of Medicine," Dr. Anselmier explained a process for the discovery of foreign bodies of ferruginous nature by the magnetic needle. He suspends to a fixed point by means of a thread without torsion, a magnetic needle, about 15 to 20 centimetres in length. When it has become perfectly motionless, the part where the foreign body is supposed to be lodged is approximated to one of the poles; the deviation or immobility of the needle, will render the diagnosis certain.

M. Boinet commends iodine highly as a preventive and cure for purulent infection. He concludes his paper to this effect: "Iodine, or its preparations, by its specific action, particularly upon inflamed tissues, is not only a remarkable antiseptic, but also a veritable antiphlogistic, which has the property, in modifying the inflamed surfaces, of diminishing, arresting, and dispersing inflammation. It acts thus in a great number of inflammatory cutaneous affections, in erysipelas, angioleucitis, in the pustules of variola, where it suffices to abort or make those inflammations disappear, to practice a few paintings of tr. iodine with a brush upon the skin or mucous membrane inflamed."

Auscultation of the head from the analysis of 300 observations made by Dr. Roger, although barren of results for the diagnosis of cerebral disease, is likely to render unlooked-for service in the diagnosis of alterations of the blood.

M. Deleau has found perchloride of iron efficacious in spermatorrhœa; he applies it in the form of a pomade to the scrotum, and administers it internally in the form of syrup.

Respecting the use of perchloride of iron in diphtheria, Dr. Isnard remarks: "The perchloride of iron ought to be administered, the nearest possible to the commencement of the affection, and in high doses; its employment should be continued at all periods of the disease, both when the false membranes are formed, and when the general infection is declared. At all these times, its action will be the same,—more physico-chemical upon the elements of the blood and the respiratory mucous membrane, than dynamic upon the nervous system."

Dr. Pirplu has treated successfully a case of vesical catarrh by injections of tannin, commencing with about gr. xv. to half a glass of water, and gradually increasing the quantity. Immediate relief followed the first enema.

*Lavements* of wine, per rectum, combined with opium, were employed with entire success in an apparently moribund condition from post-partum hemorrhage.

In an epidemic of gangrenous angina, at Bayonne, M. Silva has derived the happiest results from the local application of iodine.

Dr. Roche claims to have obtained great advantages for the past two years, in the treatment of the severer forms of angina, from irrigations of solution of common salt. The solution is rendered saline up to the point that it can be drunk without disgust. An irrigator (*Egnisier's*) furnished with a very fine jet, is employed to keep up a continual stream every hour. The patient sits or reclines upon the side, the body leaning over a basin to receive the liquid in proportion as it flows from the mouth. It is worthy of notice that in the reported cases both chlorate of potass and cauterization with arg. nit. were also employed.

At the clinique (*Hôp. de la Charité*), M. Beau advances the theory that exuberance of fibrine in the blood, renders that fluid irritating to the vessels carrying it, and to the parts in which it may be extravasated, in an analogous manner as the secretions in coryza inflame the upper lip, and in dysentery the skin of the anal region.

Among the ancients, inflammation was the consequence either of an excess of youthfulness or health; and a man never was so near an attack of inflammation as when he found himself in robust health. It is now manifest that a certain degree of globulic anæmia is the ordinary condition for development of the most acute phlegmasiæ, as well as of a crowd of other maladies, in hindering the anæmic individual from resisting the occasional or specific cause of the inflammation. Clinical observation and physiological experiment demonstrate this great pathological truth, "*That a certain degree of weakness or anæmia of globules is an habitual predisposing cause of inflammation.*"

Modern observation has decided that we do not "*throttle* inflammation" by venesection. Bleeding acts at first in the phlegmasiæ as in all diseases and in the healthy state, by depriving the blood of a part of its globules. This spoliation of the globules results in palor, laxity, and feebleness, which deprive the organism of the resources necessary to bring about resolution. Bleeding increases the quantity of fibrine. As this ingredient is already augmented, it follows that bleeding, when inflammation *already exists*, aggravates the evil. The good blood is abstracted and the bad blood is increased. M. Beau concludes that bleeding, as a curative and ectrotic, should be proscribed; yet, employed with great discretion, it may be retained in our therapeutics,—as a palliative in rare cases.

